# Opioid-Free Anesthesia for Mandibular Surgery in a Patient With Confirmed Opioid Hypersensitivity: A Case Report

**DOI:** 10.7759/cureus.102932

**Published:** 2026-02-03

**Authors:** Adam M Roumani, Souad Chahboune, Toufik Iaiche-Achour

**Affiliations:** 1 Faculty of Medicine, University of Health Sciences, Algiers, DZA

**Keywords:** dexmedetomidine, opioid allergy, opioid-free general anesthesia, oral and maxillofacial surgery, perioperative pain management

## Abstract

Opioid-induced anaphylaxis is a rare but potentially life-threatening condition that represents a major challenge for perioperative pain management. We report the case of a 38-year-old woman with confirmed hypersensitivity to fentanyl and sufentanil, who underwent mandibular cyst resection under exclusive systemic opioid-free anesthesia. Following a previous aborted surgery due to an anaphylactic reaction during induction, allergy testing confirmed opioid hypersensitivity, while other intravenous anesthetics tested negative. Anesthesia was performed using a multimodal systemic approach combining dexmedetomidine, ketamine, lidocaine, magnesium sulfate, dexamethasone, and paracetamol, without regional anesthesia. Intraoperative hemodynamic parameters remained stable, and no allergic or anesthetic complications occurred. Postoperative pain was acceptable and managed with non-opioid analgesics, and patient satisfaction was high. This case highlights the feasibility of a fully systemic opioid-free anesthetic strategy in maxillofacial surgery for patients with confirmed opioid allergy.

## Introduction

The fear of postoperative pain is the most important risk factor for perioperative patient anxiety [1]. Approximately 80% of patients report moderate to severe pain following maxillofacial surgery [2]. Opioids are the cornerstone of pain management during surgery [3]. However, in rare cases (1.7%), patients may present IgE-mediated opioid hypersensitivity, which precludes the use of opioids during surgery [4].

Opioid-free anesthesia (OFA) is a multimodal analgesic and anti-hyperalgesic approach, developed as an alternative to avoid opioid-related adverse effects, particularly postoperative nausea and vomiting (PONV) [5]. Most OFA protocols incorporate regional anesthesia as an adjuvant opioid sparing strategy [6,7]. However, data regarding total OFA without regional nerve blocks in maxillofacial surgery remain limited.

We report the case of a patient with confirmed opioid hypersensitivity, who successfully underwent mandibular cyst resection surgery using a systemic multimodal opioid-free anesthetic approach.

## Case presentation

We present the case of a 38-year-old woman scheduled for the resection of a mandibular cyst. The patient had previously undergone an attempted surgical procedure in another institution. During anesthesia induction, she developed a severe anaphylactic reaction, leading to immediate discontinuation of anesthesia and cancellation of the surgery. She was subsequently referred to a specialized allergy center where skin prick testing confirmed hypersensitivity to fentanyl and sufentanil. Skin tests performed with propofol, rocuronium, and latex were negative. No specific IgE assays were performed.

The remainder of the patient’s medical history was normal, and no other drug or food allergies were reported. Preoperative evaluation, including vital signs, systemic examination, and laboratory investigations, revealed no abnormalities. Preoperative airway assessment did not identify any predictors of difficult airway management. The Apfel score of the patient was 2/4.

Regional anesthesia techniques were considered as part of a multimodal OFA strategy. However, due to local organizational constraints and limited availability of expertise in ultrasound-guided regional anesthesia for maxillofacial surgery, no regional block was performed. Consequently, a systemic multimodal analgesic approach was selected and implemented.

Upon arrival in the operating room, standard monitoring, including continuous electrocardiography, non-invasive blood pressure, and pulse oximetry, was applied. The patient presented with marked tachycardia, with a heart rate of 140 bpm in the absence of other clinical abnormalities, likely related to significant preoperative anxiety. Intravenous preemptive analgesia consisting of 1 g paracetamol, 20 mg nefopam, 1.5 g magnesium sulfate, and 12 mg dexamethasone was administered over 10 minutes. An induction dose of dexmedetomidine (0.25 µg/kg) was administered over five minutes.

After three minutes of preoxygenation with 100% oxygen via a tightly fitted face mask, a sequential intravenous induction of anesthesia was initiated with propofol (3 mg/kg), dexmedetomidine (0.1 µg/kg), ketamine (0.25 mg/kg), lidocaine 2% (1.6 mg/kg), and rocuronium (0.6 mg/kg)

Laryngoscopy using a Besdata® videolaryngoscope with a Macintosh 3 blade revealed a Cormack-Lehane grade I view. Following successful nasotracheal intubation, ketamine (0.5 mg/kg) was given to provide analgesic coverage for surgical incision, dexamethasone 8 mg for PONV prophylaxis, and antibiotic prophylaxis with 2 g cefazolin and 500 mg metronidazole was given. No other antiemetic drugs were available at the time of surgery.

Anesthesia was maintained with sevoflurane, in combination with continuous infusion of dexmedetomidine (0.1 µg/kg/h), ketamine (0.2 mg/kg/h), and lidocaine 2% (1.6 mg/kg/h). Prior to incision, local infiltration of the jugal mucosa with 1% lidocaine with epinephrine was performed by maxillofacial surgeons.

**Figure 1:**
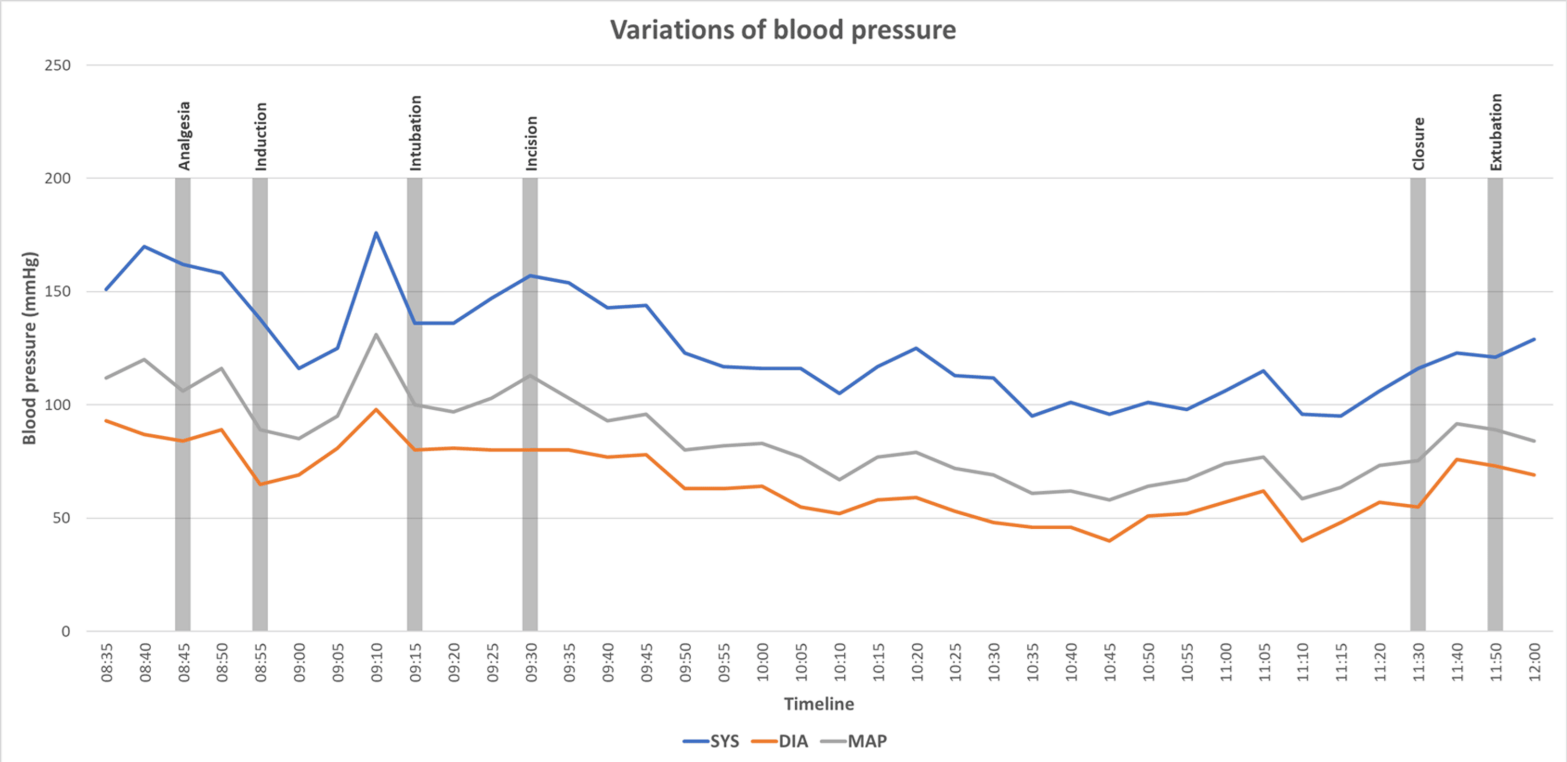
Variation of blood pressure during the intervention

**Figure 2:**
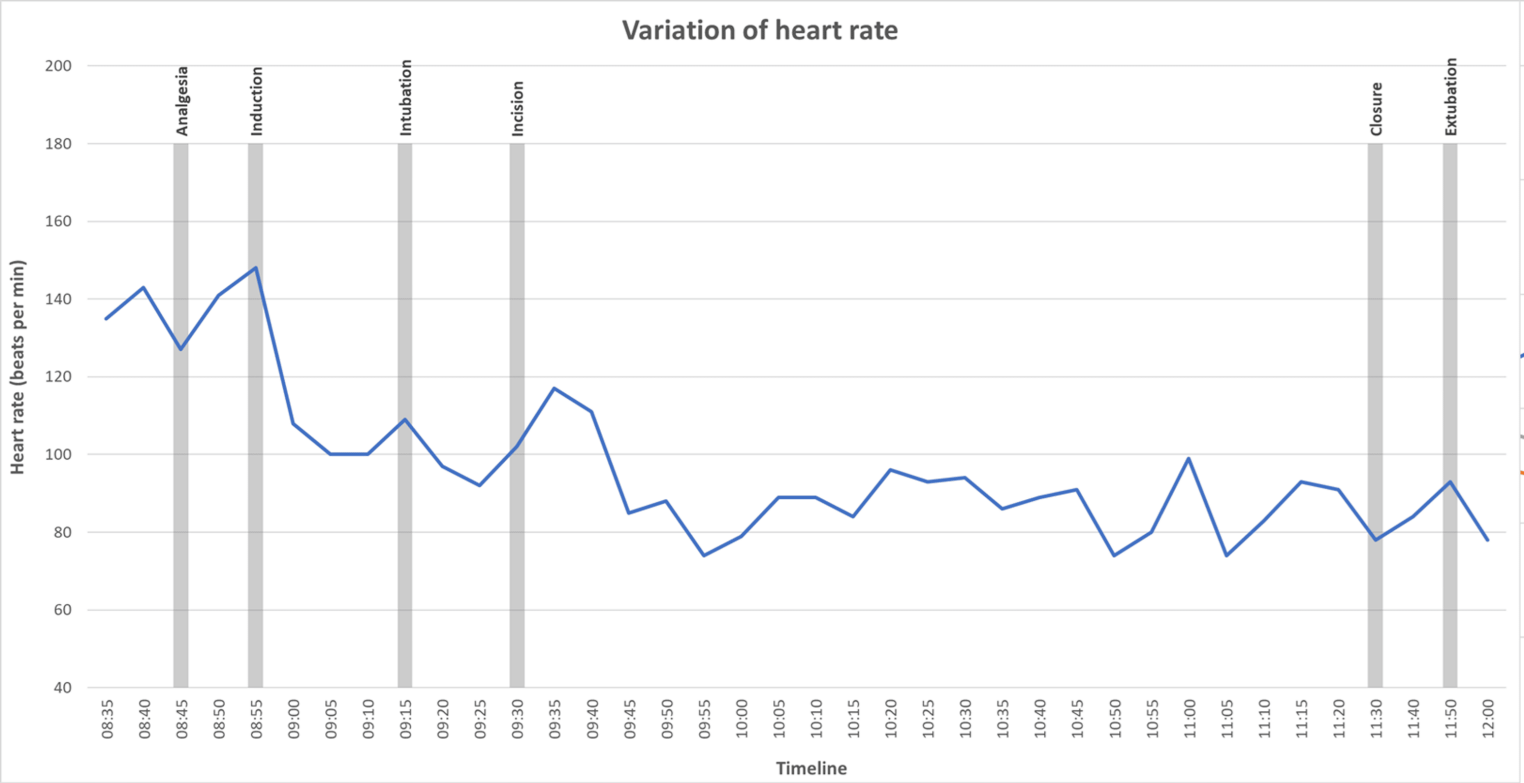
Variation of heart rate during the intervention

Hemodynamic parameters remained stable throughout the perioperative period. No episode of hypotension (MAP < 60 mmHg) or bradycardia (HR < 45 bpm) requiring pharmacological intervention was observed. A graphical representation of heart rate and blood pressure during the different anesthetic and surgical stages is provided in Figure 1 and Figure 2.

Continuous infusions of dexmedetomidine, ketamine, and lidocaine were discontinued 15 minutes prior to extubation. The total intraoperative doses of dexmedetomidine, ketamine, and lidocaine were 17.8 µg, 36.6 mg, and 292 mg, respectively. Paracetamol 1 g was administered for postoperative analgesia. Neuromuscular blockade was reversed with sugammadex (100 mg), and intravenous lidocaine 1% (100 mg) was given to blunt the coughing reflex during emergence.

Awakening and extubation were smooth and uneventful. The patient was subsequently transferred to the inpatient ward. The immediate postoperative Numeric Pain Rating Scale (NPRS) was < 3. The patient reported two episodes of PONV during the first 24 hours. NPRS at H3 and H12 was 5/10, successfully managed by Paracetamol 1 g administration. The patient reported high satisfaction with the anesthetic and surgical management.

## Discussion

This case shows the feasibility of a systemic multimodal opioid-free anesthetic strategy for a patient with confirmed hypersensitivity to fentanyl and sufentanil undergoing maxillofacial surgery. Despite the high-risk allergic context and the significant preoperative anxiety, anesthesia was conducted uneventfully without opioids and without regional anesthesia, with satisfactory intraoperative hemodynamic stability and acceptable postoperative analgesia.

Perioperative anaphylaxis is a rare but potentially life-threatening event, most commonly triggered by neuromuscular blocking agents (58.1%), latex (19.7%), and antibiotics (12.9%) [4]. Most adverse reactions to opioids are related to pseudo-anaphylactic reactions, rather than true IgE-mediated allergies, with a low prevalence of cross-reactivity [8]. In this case, the diagnosis of fentanyl and sufentanil hypersensitivity was confirmed by skin prick testing, which led to the adaptation of the anesthetic management, despite the absence of a specific IgE assay.

OFA has gained increasing popularity with the emergence of dexmedetomidine as an analgesic and hypnotic agent, particularly when combined with regional anesthesia [9]. However, few studies in the literature have reported OFA protocols specifically applied to maxillofacial surgery. In contrast, opioid-sparing strategies aimed at reducing opioid-related adverse effects are well documented [10,11]. The combination of dexmedetomidine, ketamine, and lidocaine provides complementary and synergistic antinociceptive effects, while contributing to hemodynamic stability and reducing perioperative opioid requirements [5].

The originality of this case lies in the successful use of a systemic OFA protocol for a patient with confirmed opioid hypersensitivity undergoing maxillofacial surgery. Although pain scores were mildly elevated in the first postoperative hours, they were successfully managed with first-line analgesia, and patient satisfaction remained high.

This report presents some limitations. As a single case, the results cannot be generalized, compromising the external validity. The absence of regional anesthesia and the lack of specific IgE assays may limit the interpretation of both analgesic efficacy and allergic assessment. Additionally, postoperative analgesia and PONV prevention were not optimal, suggesting the need for individualized strategies and improved multimodal approaches. Further studies are required in order to better define the role of OFA protocols in maxillofacial surgery, particularly in patients with contraindications for opioid use.

## Conclusions

This case demonstrates that exclusive systemic multimodal OFA is a feasible option for maxillofacial surgery in patients with confirmed opioid-induced anaphylaxis, even in the absence of regional anesthesia. Careful perioperative planning remains essential to ensure safety and adequate analgesia.
